# A logistic-tent chaotic mapping Levenberg Marquardt algorithm for improving positioning accuracy of grinding robot

**DOI:** 10.1038/s41598-024-60402-1

**Published:** 2024-04-26

**Authors:** Jian Liu, Yonghong Deng, Yulin Liu, Linlin Chen, Zhenzhen Hu, Peiyang Wei, Zhibin Li

**Affiliations:** 1 School of Economics and Management, Chengdu Technological University, Chengdu, 611730 Sichuan China; 2 Sichuan Institute of Industrial Big-Data Applications, Chengdu, 611730 China; 3 School of Software Engineering, Chengdu University of Information Technology, Chengdu, 610225 China; 4 College of Communication Engineering, Chengdu University of Information Technology, Chengdu, 610225 China; 5 School of Computer Science and Technology, Chongqing University of Posts and Telecommunications, Chongqing, 400065 China

**Keywords:** Grinding robot, Geometric error identification and compensation, Positioning accuracy, Logistic-tent chaotic mapping, Levenberg–Marquardt, Mechanical engineering, Applied mathematics

## Abstract

The precision of workpiece machining is critically influenced by the geometric errors in the kinematics of grind robots, which directly affect their absolute positioning accuracy. To tackle this challenge, this paper introduces a logistic-tent chaotic mapping Levenberg Marquardt algorithm designed to accurately identify and compensate for this geometric error. the approach begins with the construction of a forward kinematic model and an error model specific to the robot. Then the algorithm is adopted to identify and compensate for the geometric error. The method establishes a mapping interval around the initial candidate solutions derived from iterative applications of the Levenberg Marquardt algorithm. Within this interval, the logistic-tent chaotic mapping method generates a diverse set of candidate solutions. These candidates are evaluated based on their fitness values, with the optimal solution selected for subsequent iterations. Empirical compensation experiments have validated the proposed method's precision and effectiveness, demonstrating a 6% increase in compensation accuracy and a 47.68% improvement in efficiency compared to existing state-of-the-art approaches. This process not only minimizes the truncation error inherent in the Levenberg Marquardt algorithm but also significantly enhances solution efficiency. Moreover, simulation experiments on grind processes further validate the method's ability to significantly improve the quality of workpiece machining.

## Introduction

The industrial robot is a crucial production equipment that incorporates various modern industrial technologies, such as mechanical manufacturing, computer processing, and information interaction. It finds extensive application in the production of high-precision parts, including the grinding of large impellers, the welding of aircraft skins, the polishing and grinding of precision components, and more^1–5^. A key aspect of robot functionality, particularly in grinding operations, is the force control of the end effector, which is essential for maintaining a consistent grinding force. However, challenges arise when a robot's positioning accuracy is suboptimal, leading to fluctuations in the grinding tool's position relative to the workpiece during machining. Such positional variability can introduce inconsistencies in grinding force, adversely affecting the uniform removal of material from the workpiece and, consequently, the quality of the finished product. Addressing this issue, enhancing the robot's absolute positioning accuracy through kinematic compensation of geometric parameters becomes critical. By refining these parameters, robots can achieve a stable grinding tool pressure at the end effector, ensuring a uniform grinding force. This approach emphasizes the significance of precision in the robot's positioning capabilities to maintain consistent operational performance. Despite the robot achieving a repeatability in positioning accuracy of 0.01 mm, its absolute positioning accuracy is still limited. This limitation makes it challenging to meet the precision requirements for manufacturing high-precision parts. The main reason for poor absolute positioning accuracy is kinematic error, which result from joint sensor error, geometric error, and non-geometric error. Geometric error contributes to more than 80% of the total error, directly impacting the robot's running accuracy^1^.

Densifying and compensating for the geometric parameters of a robot is an effective and cost-efficient approach to enhance its absolute positioning accuracy^2^. The compensation process involves a four-step procedure: kinematic error modeling, end position measurement, parameter identification, and error compensation. Widely adopted geometric parameter models in robotics include the Denavit-Hartenberg (DH) model^3^ and the modified DH (MDH) model^4^, along with the complete and parametrically continuous (CPC) Model^5^, the exponential product (POE) model^6^, and the Stone (S) Model^7^. The challenge in robotic error modeling lies in its high-dimensional multiparameter and strong nonlinearity characteristics, making the identification of geometric errors a task of optimizing nonlinear functions. Despite the application of various methods such as the least square method^8^, maximum likelihood method^9^, extended Kalman filtering method^10^, and LM method^11^ to solve these models, the accuracy of their solutions remains constrained.

Recognizing the limitations inherent in these traditional methods and the complexity of robotic error models, researchers have been motivated to explore more advanced calibration techniques. This pursuit led to the development of innovative solutions such as the self-calibration method for dual-manipulators proposed by Zhu et al.^12^, which leverages the particle swarm optimization algorithm, demonstrating a significant enhancement in the positioning accuracy of two robots. Similarly, Le et al.^13^ introduced a robotic calibration algorithm employing an artificial neural network and invasive weed optimization, with experimental validations confirming the method's efficacy. Further contributions include Yan et al.^14^ applied a genetic algorithm to refine the accuracy of a 6-DOF parallel robot and Jiang et al*.*^15^ used extended Kalman filter and particle filter algorithms to calibrated kinematic parameter, both achieving notable improvements in absolute positioning accuracy. Deng et al.^16^ proposed a hybrid algorithm combining the Levenberg–Marquardt (LM) algorithm with an opposition-based learning squirrel search algorithm for identifying the kinematic parameters of a polishing robot, achieving a 62.61% improvement in absolute positioning error following calibration. Bastl et al.^17^ introduced a calibration technique using a multi-objective deep learning evolutionary algorithm and a reference vector-based evolutionary algorithm to improve robot accuracy, showing effectiveness in dealing with noisy data. *Chen *et al.^18^ introduced a kinematic calibration method utilizing an improved beetle swarm optimization algorithm, enhanced by a preference random substitution method for industrial robots, significantly improving positioning accuracy in drilling and riveting tasks, with experiments demonstrating a reduction in end-effector position error from 2.95 mm to 0.20 mm. Li et al.^19^ developed a novel calibration algorithm combining an unscented Kalman filter with a variable step-size LM method for industrial robots, significantly enhancing calibration accuracy and outperforming state-of-the-art methods in empirical studies. Xu et al.^20^ introduced an enhanced manta ray foraging optimization algorithm for calibrating kinematic parameters of robotic arms, significantly reducing positioning errors through efficient identification and adjustment of parameter inaccuracies. However, these innovative approaches, while effective to some extent, encounter limitations such as slow computational speed, low efficiency, and inadequate accuracy, highlighting the ongoing challenge of satisfying the rigorous accuracy demands for solving complex convex optimization problems.

The LM algorithm, known for its computational simplicity and efficiency, is a commonly used approach for identifying robotic geometric error. It combines Newton's method with the steepest descent method and is an improved version of the Least Squares algorithm, renowned for its robustness, fast convergence, and strong local optimization capabilities. The LM algorithm combines the advantages of Newton's method and the steepest descent method. Therefore, it is a common technique for robot error identification^21–23^. Despite its high computational efficiency and solution accuracy, the LM algorithm is prone to truncation error due to its reliance on the first-order Taylor expansion approximation. Specifically, the algorithm tends to experience stagnation when the search approaches the optimal solution.

This paper proposes a logistic-tent chaotic mapping Levenberg Marquardt algorithm (LTLM) to enhance the accuracy of the LM algorithm in solving grinding robot error models. The main contributions of this study are:

- The logistic-tentative chaotic mapping is integrated into the update rule of the standard LM algorithm to obtain an LTLM algorithm with faster convergence speed and higher identification accuracy.
- The algorithm design is meticulously implemented, and its concise code serves as a valuable reference for scholars and engineers seeking to implement it.
- Presenting a comprehensive analysis of compensation techniques and conducting simulation machining experiments to offer a viable approach for enhancing the machining accuracy of ultra-precision components using smooth robots.

The experimental results demonstrate that it has a superior convergence speed and higher convergence accuracy in solving robot error models when compared with the state-of-the-art compensation algorithms.

The organization of this paper is as follows: the kinematic and error Models are established in Section "Grinding robot kinematic and error models". In Section "The LTLM algorithm for geometric error identification", a LTLM algorithm is proposed to identify geometric error. In Section "Compensation performance evaluation", compared with other algorithms, the performance of the LTLM algorithm is analyzed. Section "Grinding compensation experiment" presents the grinding polishing simulation experiments. The conclusions are presented in Section "Conclusion".

## Grinding robot kinematic and error models

### Introduction of grinding robot system

**Figure 1:**
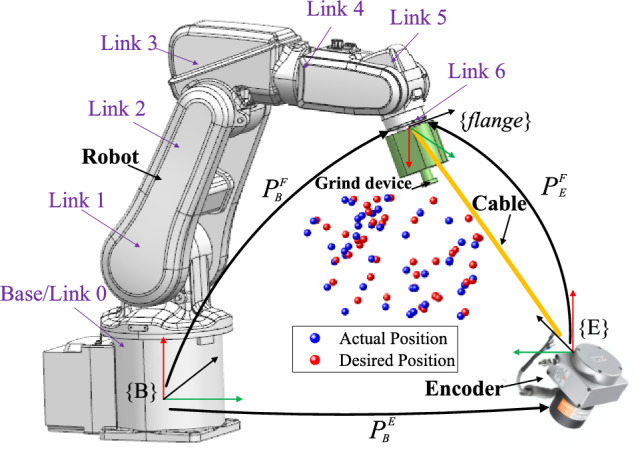
Grinding robot system and measuring devices.

Figure 1 depicts the grinding robot system and measuring devices. The grinding robot system comprises an ABB IRB120 industrial robot with six revolute joints, grinding device and worktable. Measuring devices include a wire-draw-encoder, a cable length display, and a computer running relevant application software. During machining operations, the grinding device is mounted on the robot's end flange. Neglecting installation error allows for the identification of geometric error in the robot's kinematic joints. Hence, when measuring the spatial position of the grinding robot, the grinding device is detached, and the wire-draw-encoder measuring end is affixed to the central area of the flange end face for accurate measurement.

Figure 1 illustrates the relevant coordinate systems in the system. {B} refers to the robot base coordinate system, {*flange*} is the coordinate system of the flange center point at the end of the robot, and {E} is the coordinate system of the wire-draw-encoder outlet port. Each coordinate system has the following relationship:

1 $$L_{E}^{F} = \left\| {{\mathbf{P}}_{B}^{F} - {\mathbf{P}}_{B}^{E} } \right\|$$

where **L***F E* is the nominal cable length. **P***F B* is the calculated position coordinate value from {B} to {*flange*}, which includes the geometric error that need to be identified. **P***E B* is the position coordinate value from{B} to {E}, which can be directly measured by wire-draw-encoder.

### Kinematic and error models

The most commonly used method for modeling robot kinematics is the Denavit-Hartenberg (D-H) method, first proposed by Denavit and Hartenberg^24^. This method utilizes the geometric parameters of all the robot's freedom joints to determine its end position and posture. The robot's structure is defined by four parameters for each joint and connecting link based on the structural parameters and the kinematic form between adjacent links. The nominal D-H parameters of the ABB IRB120 industrial robot are listed in Table 1. In D-H model, *a*, *d*, *α* and *θ* are the link length, the link offset distance, the link twist angle and the joint angle, respectively. The pose of the *i*-th joint coordinate system relative to the *i*-1-th joint coordinate system can be uniquely determined using four mutually independent parameters, which are defined for each joint and connecting link. This is achieved through the determined homogeneous transformation method^8^.

**Table 1:** The nominal D-H parameters for ABB IRB120.

Joint *i*	*a*_*i*_*/*mm	*d*_*i*_*/*mm	*α*_*i*_*/*°	*θ*_*i*_*/*°
1	0	290	− 90	0
2	270	0	0	− 90
3	70	0	− 90	0
4	0	302	90	0
5	0	0	− 90	0
6	0	72	0	0

The homogeneous transformation relationship matrix of adjacent joint coordinate systems can be expressed as follows:

2 $${\mathbf{T}}_{i}^{i - 1} = \left[ {\begin{array}{*{20}c} {\cos \theta_{i} } & { - \sin \theta_{i} \cos \alpha_{i} } & {\sin \theta_{i} \sin \alpha_{i} } & {a_{i} \cos \theta_{i} } \\ {\sin \theta_{i} } & {\cos \theta_{i} \cos \alpha_{i} } & { - \cos \theta_{i} \sin \alpha_{i} } & {a_{i} \sin \theta_{i} } \\ 0 & {\sin \alpha_{i} } & {\cos \alpha_{i} } & {d_{i} } \\ 0 & 0 & 0 & 1 \\ \end{array} } \right]$$

where **T***i-*1* i* is the transformation matrix from *i*-1-th link to *i*-th link,* a*_*i*_, *d*_*i*_, *α*_*i*_ and *θ*_*i*_ represent the link length, the link offset distance, the link twist angle and the joint angle of the *i*-th link, respectively.

According to Eq. (2), the forward kinematic model of the robot with six joints is calculated as follows:

3 $${\mathbf{T}}_{6}^{0} = \prod\limits_{i = 1}^{6} {{\mathbf{T}}_{i}^{i - 1} } = \left[ {\begin{array}{*{20}c} {{\mathbf{R}}_{N} } & {{\mathbf{P}}_{N} } \\ 0 & 1 \\ \end{array} } \right]$$

where **R**_*N*_ stands for rotation matrix, **P**_*N*_ denotes the position vector. Thereby, the position of the robot end-effector relative to the base coordinate system can be obtained.

When there are deviations in the D-H parameters, based on Eq. (3), it can be obtained that:

4 $${\mathbf{T}}_{6}^{0} + \delta {\mathbf{T}}_{6}^{0} = \prod\limits_{i = 1}^{6} {({\mathbf{T}}_{i}^{i - 1} + } \delta {\mathbf{T}}_{i}^{i - 1} )$$

Consequently, the differential transformation matrix for each link is expressed as follows:

5 $$\delta {\mathbf{T}}_{i}^{i - 1} = \frac{{\partial {\mathbf{T}}_{i}^{i - 1} }}{{\partial a_{i} }}\delta a_{i} + \frac{{\partial {\mathbf{T}}_{i}^{i - 1} }}{{\partial d_{i} }}\delta d_{i} + \frac{{\partial {\mathbf{T}}_{i}^{i - 1} }}{{\partial \alpha_{i} }}\delta \alpha_{i} + \frac{{\partial {\mathbf{T}}_{i}^{i - 1} }}{{\partial \theta_{i} }}\delta \theta_{i}$$

By expanding Eq. (4) and ignoring the high-order differential terms can be achieved:

6 $$\delta {\mathbf{T}}_{6}^{0} = \sum\limits_{i = 1}^{6} {({\mathbf{T}}_{1}^{0} {\mathbf{T}}_{2}^{1} \cdots {\mathbf{T}}_{i - 1}^{i - 2} } \delta {\mathbf{T}}_{i}^{i - 1} {\mathbf{T}}_{i + 1}^{i} \cdots {\mathbf{T}}_{5}^{4} {\mathbf{T}}_{6}^{5} )$$

Hence, the mapping relationship between position error and parameter error can be depicted as:

7 $$\delta {\mathbf{P}} = \left[ {\begin{array}{*{20}c} {{\mathbf{J}}_{a} } & {{\mathbf{J}}_{d} } & {{\mathbf{J}}_{\alpha } } & {{\mathbf{J}}_{\theta } } \\ \end{array} } \right]\left[ {\begin{array}{*{20}c} {\delta {\mathbf{a}}} \\ {\delta {\mathbf{d}}} \\ {\delta {{\varvec{\upalpha}}}} \\ {\delta {{\varvec{\uptheta}}}} \\ \end{array} } \right] = {\mathbf{J}}{\mathbf{x}}$$

where δ**P** indicates the position error vector of robot end, **J** denotes the Jacobian matrix, **x** is the geometric error vector.

The least square objective function *f* can be constructed:

8 $$f = \arg \mathop {\min }\limits_{{\mathbf{x}}} \frac{1}{2}\left\| {\delta {\mathbf{P}}} \right\|_{2}^{2} = \min \left[ {\frac{1}{n}\sum\limits_{i = 1}^{n} {\frac{1}{2}(l_{i} - l_{i}^{*} )^{2} } } \right]$$

where *n* represents the number of sample points. l* i denotes the measuring cable length. *l*_*i*_ is the nominal cable length, which can be calculated from the robot end position **P**_*i*_, where **P**_*i*_ is **P***F B* in Eq. (1).

9 $$l_{i} = \sqrt {({\mathbf{P}}_{i} - {\mathbf{P}}_{B}^{E} )^{2} }$$

To achieve the highest positioning accuracy, it is important to minimize the objective function by determining the kinematic parameters of the robot that are closest to their actual values. This can be achieved by accurately solving the model to reduce the deviations between the nominal and actual kinematic parameters. Clearly, *f* is a transcendental equation, and traditional methods cannot yield an analytical solution. To overcome this limitation, we employed the LTLM algorithm as a novel approach to address this issue.

## The LTLM algorithm for geometric error identification

### LM algorithm for identification

The LM algorithm is a hybrid of the Newton-Gauss and steepest descent methods, which leverage their individual strengths^25,26^. By addressing the shortcomings of the Gauss–Newton method, it is more robust. The algorithm updates the current position in the gradient descent direction and iteratively cycles until an optimal solution is found. It is well-suited for solving the optimal problem of the nonlinear multivariate objective function and can resolve non-positive definite and singular Hessian construction matrix problems, thus being effective in solving robot error models.

The iteration form of the standard LM algorithm is as follows:

10 $${\mathbf{x}}_{k + 1} = {\mathbf{x}}_{k} - ({\mathbf{J}}_{k}^{T} {\mathbf{J}}_{k} + \mu I)^{ - 1} {\mathbf{J}}_{k}^{T} f({\mathbf{x}}_{k} )$$

where *f*(•) indicates the objective function given by Eq. (8), *f*(**x**_*k*_) represents the position error at the *k-*th iteration, and its size is *n* × 1. **x**_*k*_ is the deviation vector of the geometric parameters at the *k-*th iteration, its size is 24 × 1. **J**_*k*_ denotes the Jacobian matrix at the *k-*th iteration, which is *n* × 24 vector. *µ* is the damping factor, which is taken as 5 in this paper.

The iteration loop concludes either when the iteration achieves the desired accuracy or when the maximum number of iterations is reached. The LM algorithm employs a first-order Taylor series approximation, leading to the presence of inevitable truncation error at each iteration step.

### The LTLM algorithm for identifying robot geometric error

To reduce the truncation error of LM, logistic-tent chaotic mapping is adopted to address this issue. In optimization, chaotic mappings offer a compelling alternative to pseudo-random numbers, enhancing algorithm performance through improved diversity and exploration, preventing premature convergence to local optima^27–29^. Table 2 outlines the merits and drawbacks of various chaotic mappings, guiding the selection for optimization tasks. The Logistic-Tent chaotic mapping, combining the Logistic map's rich chaotic behavior with the Tent map's rapid iteration and adaptability, emerges as particularly beneficial^30–32^.

**Table 2:** Comparison of several chaotic mappings.

Mapping methods	Advantages	Disadvantages
Logistic mapping	Simple to implement; well-studied with a clear understanding of its chaotic dynamics	Limited range of chaotic behavior; prone to periodic windows which can reduce its effectiveness in complex optimization tasks
Tent mapping	Fast iteration speed; uniform distribution of values which is beneficial for certain types of random processes	Simplicity of the dynamics can limit its effectiveness in navigating complex solution spaces
Sine mapping	Exhibits robust chaotic behavior over its entire parameter range; easy to implement	Can be predictable under certain conditions, limiting its utility in enhancing algorithmic randomness and complexity
Chebyshev mapping	Strong chaotic behavior across its entire parameter range, useful for ensuring robust global search capability	Higher computational complexity compared to simpler maps, which may not be ideal for all applications
Henon mapping	Provides a higher dimensional chaotic sequence, which can be useful for more complex optimization problems	Increased complexity and computational requirements compared to one-dimensional maps
Bernoulli mapping	Simple and exhibits deterministic chaos, making it useful for certain types of optimization problems	Lack of variability in chaotic dynamics, which may limit its application scope

Inspired by the above, logistic-tentative chaotic mapping is introduced into the update rule of the LM algorithm to improve the identification accuracy of geometric error.

The mathematical formulation of the Logistic-Tent mapping is presented as follows:

11 $$N_{m + 1} = \left\{ {\begin{array}{*{20}c} {\bmod (rN_{m} (1 - N_{m} ) + \frac{{(4 - r)N_{m} }}{2},1),N_{m} < 0.5} \\ {\bmod (rN_{m} (1 - N_{m} ) + \frac{{(4 - r)(1 - N_{m} )}}{2},1),N_{m} \ge 0.5} \\ \end{array} } \right.$$

where *N*_*m*_ represents the *m*-th distribution point. *r* is a hyperparameter, *r* ε [0,4].

For the chaotic sequence generated under each value of *r*, we distribute the sequence into a predetermined number of bins (e.g., 50), and then calculate the standard deviation of the frequencies across these bins. The formula for standard deviation is given by:

12 $$\sigma = \sqrt {\frac{1}{N - 1}\sum\limits_{i = 1}^{N} {(c_{i} - c^{\prime})^{2} } }$$

where *c*_*i*_ represents the frequency count of the *i*-th bin, *c*´ is the average frequency count across all bins, and *N* is the total number of bins.

**Figure 2:**
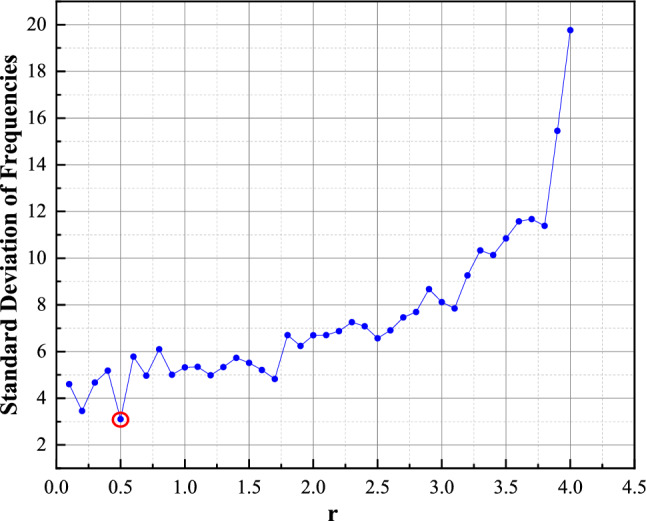
The relationship between *r* and the standard deviation of frequencies.

By varying the value of* r* (e.g., from 0 to 4), we can determine the standard deviation of frequencies associated with each *r* value. Ultimately, we plot a graph with *r* values on the horizontal axis and the corresponding standard deviation of frequencies on the vertical axis, as shown in Fig. 2. This graph allows us to observe how the uniformity of the distribution is affected by the *r* value. By comparing the standard deviations across different *r* values, we can identify the *r* value that results in the most uniform distribution, indicated by the smallest standard deviation. Our analysis has determined that *r* = 0.5 achieves the best uniformity in the distribution, making it the optimal choice for achieving a highly uniform chaotic sequence.

**Figure 3:**
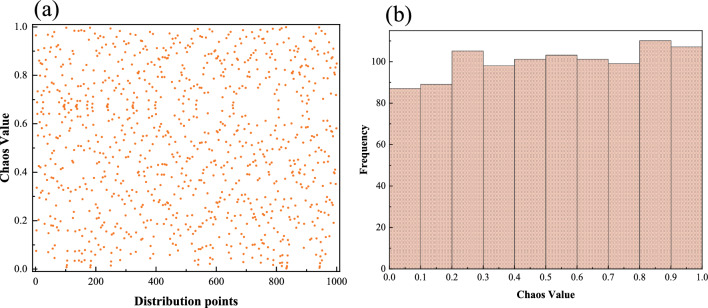
Logistic-tent chaotic mapping distribution (r = 0.5, m = 1000). (**a**) Distribution situation; (**b**) Distribution histograms.

Figure 3 depicts the distribution of Logistic-Tent chaotic mapping. Obviously, the Logistic-Tent chaotic mapping exhibits superior chaotic properties. As observed in Fig. 2a, the generation of 1,000 points is uniformly distributed across the [0,1] range, showcasing not only excellent uniformity but also remarkable randomness. This uniformity is further evidenced in Fig. 2b, where the quantity of points associated with each value within the [0,1] range shows minimal variation, indicating a high level of evenness. Hence, the Logistic-Tent chaotic mapping demonstrates outstanding randomness and distribution uniformity, affirming its superior chaotic characteristics.

Integrating the Logistic-Tent chaotic mapping into the update rules of the standard LM algorithm. The LM for two consecutive iterations results in the following outcomes:

13 $${\mathbf{x}}_{k + 1,1} = {\mathbf{x}}_{k} - ({\mathbf{J}}_{k}^{T} {\mathbf{J}}_{k} + \mu I)^{ - 1} {\mathbf{J}}_{k}^{T} f({\mathbf{x}}_{k} )$$

14 $${\mathbf{x}}_{k + 1,2} = {\mathbf{x}}_{k + 1,1} - ({\mathbf{J}}_{k,1}^{T} {\mathbf{J}}_{k,1} + \mu I)^{ - 1} {\mathbf{J}}_{k,1}^{T} f({\mathbf{x}}_{k + 1,1} )$$

Given the presence of truncation error in the LM iteration, it is evident that there exists at least one value near **x**_*k*+1,1_ that is more optimal than **x**_*k*+1,1_. This value can be found either to the left or right of **x**_*k*+1,1_, falling within left interval [**x**_*k*_, **x**_*k*+1,1_] or right interval [**x**_*k*+1,1_, **x**_*k*+1,2_]. Since this point is in close proximity to **x**_*k*+1,1_, it is possible to further narrow down the interval. That is, the left interval is [**a**_*k*+1_, **x**_*k*+1,1_] and the right interval is [**x**_*k*+1,1_, **b**_*k*+1_]. Note that:

15 $$\begin{gathered} {\mathbf{a}}_{k} = \left( {{\mathbf{x}}_{k} + {\mathbf{x}}_{k + 1,1} } \right)/2 \hfill \\ {\mathbf{b}}_{k} = \left( {{\mathbf{x}}_{k + 1,1} + {\mathbf{x}}_{k + 1,2} } \right)/2 \hfill \\ \end{gathered}$$

The candidate solution sets are constructed in two intervals using the Logistic-Tent chaotic mapping technique. The total number of candidate solutions is *M*. The *m*-th candidate solution can be represented by:

16 $${\mathbf{xl}}_{k + 1} (m) = {\mathbf{x}}_{k + 1,1} - ({\mathbf{x}}_{k + 1,1} - {\mathbf{a}}_{k + 1} )N_{m}$$

17 $${\mathbf{xr}}_{k + 1} (m) = {\mathbf{x}}_{k + 1,1} + ({\mathbf{x}}_{k + 1,2} - {\mathbf{b}}_{k + 1} )N_{m}$$

where *Nm* is a random number in [0,1].

The candidate solutions, along with **x**_*k*+1,1_, undergo evaluation to assess respectively their fitness values. The optimal value is then selected as the initial value for the subsequent iteration:

18 $${\mathbf{x}}_{k + 1} \leftarrow \min \left\{ \begin{gathered} f({\mathbf{xl}}_{k + 1} (1), \cdots ,f({\mathbf{xl}}_{k + 1} (M), \hfill \\ f({\mathbf{xr}}_{k + 1} (1), \cdots ,f({\mathbf{xr}}_{k + 1} (M), \hfill \\ f({\mathbf{x}}_{k + 1,1} ) \hfill \\ \end{gathered} \right\}$$

where *M* indicates the total number of candidate solutions.

Drawing upon the inferences mentioned earlier, we present a detailed workflow of Algorithm I. LTLM-GPI, which utilizes the LTLM approach for accurate identification of geometric error.
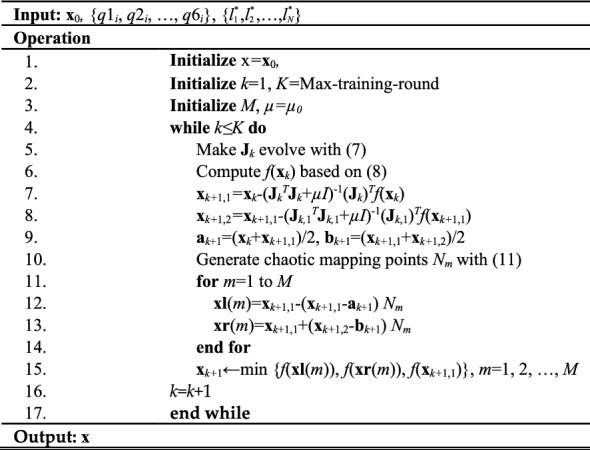


### analysis for convergence and stability of the LTLM algorithm

The LM algorithm inherently possesses convergence and stability characteristics. Upon executing a single iteration of the LM algorithm, the resultant (**x**_k+1,1_) is recognized as an approximate solution, primarily due to the presence of truncation errors. Consequently, it is posited that a solution exhibiting closer proximity to the true value inherently exists either to the left or right of (**x**_k+1,1_). To identify this more accurate solution, iteration from (**x**_k+1,1_) yields (**x**_k+1,2_), thereby situating the closer approximation within the intervals [**x**_k_, **x**_k+1,1_] or [** x**_k+1,1_, **x**_k+1,2_]. Given that the solution more closely approximating the true value is expected to be near **x**_k+1,1_, the search interval can be further narrowed to [**a**_k+1_, **x**_k+1,1_] or [**x**_k+1,1_, **b**_k+1_].

Utilizing the Logistic-Tent chaotic mapping, *M* candidate solutions are generated within these condensed intervals. Through the employment of the objective function, the optimal solution among these candidates is selected. Consequently, each iteration of the LTLM algorithm builds upon the foundational principles of the LM algorithm, thereby inheriting its convergence and stability properties. This methodology ensures that the LTLM algorithm enhances the precision of solution approximation by iteratively refining the search interval and selecting the most accurate solutions based on the defined criteria.

## Compensation performance evaluation

### Compensation process

**Figure 4:**
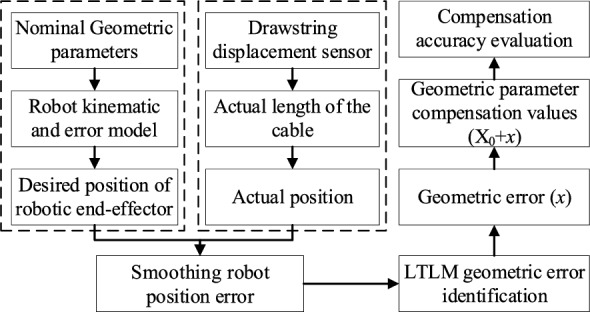
Flowchart of LTLM compensation.

To validate the effectiveness and correctness of LTLM algorithm for robot geometric error identification and compensation, the compensation valuation is conducted based on the established robot kinematic model in Section "Grinding robot kinematic and error models". The procedure chart for its compensation, as shown in Fig. 4.

As shown in Fig. 3, by adding the identified geometric parameter errors to the nominal geometric parameters, we effectively achieve compensation for the geometric parameters, as expressed in the following:

19 $$X^{*} = X_{0} + x$$

where X* is the nominal geometric parameters. *x* denotes the identified geometric error.

### Performance analysis

#### General settings

**Figure 5:**
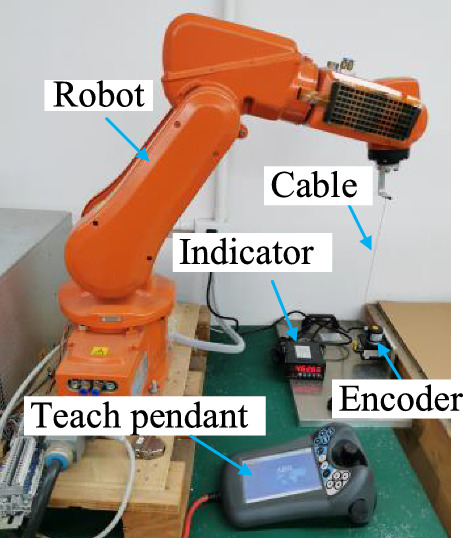
Measurement of positioning accuracy.

- (1) ***Evaluation metrics***: To evaluate the effectiveness of the compensation method in improving robot positioning accuracy, the maximum error (Max), standard deviation (STD), and root mean squared error (RMSE) as evaluation metrics are utilized to analyze the performance of those models^16–20,33^. They are:
  20 $$\begin{gathered} Max = \mathop {\max }\limits_{1 \le i \le n} \left\{ {\sqrt {(l_{i} - \hat{l}_{i} )^{2} } } \right\} \hfill \\ STD = \frac{1}{n}\sum\limits_{i = 1}^{n} {\sqrt {(l_{i} - \hat{l}_{i} )^{2} } } \hfill \\ RMSE = \sqrt {\frac{1}{n}\sum\limits_{i = 1}^{n} {(l_{i} - \hat{l}_{i} )^{2} } } \hfill \\ \end{gathered}$$
- (2) ***Dataset***: Fig. 5 depicts the measurement of the positioning accuracy of the robotic grinding system. The evaluation dataset provides pertinent parameters for an ABB IRB120 industrial robot, comprising 1024 samples. The sampling points evenly distributed in the robot's workspace. Their positions are measured using a wire-draw-encoder, while the cable length for the measuring points is displayed. Each sample data includes the robot joint kinematic angles *q*_1_ ~ *q*_6_ and the measured cable length l^*^. Five detailed samples as shown in Table 3. Note that the test data is made available at the GitHub. (https://github.com/Lizhibing1490183152/RobotCali) Table 4 provides the parameters of the cable encoder. A subset of 300 samples is randomly and uniformly selected, with a split ratio of 90% for training and 10% for testing, constituting a single test case. This procedure is replicated 10 times to generate 10 distinct test cases, the results of which are averaged to serve as the performance metric to reduce data bias. To mitigate any potential data bias, we create ten distinct data cases by randomly selecting 200 samples from a uniform distribution to generate testing data. This process is repeated ten times to ensure variability. Each data case undergoes evaluation using an 80%-20% training–testing setting to validate the tested models. The objective results are reported by recording the averaged final outcomes along with their respective standard deviations for each model.
- (3) ***Compared methods***: The performance of the LTLM method is validated by comparing it with several state-of-the-art identification methods. The methods involved in this comparison are listed in Table 5.

**Table 3:** Five detailed samples in the sample size.

NO	*q*_1_*/*°	*q*_2_*/*°	*q*_3_*/*°	*q*_4_*/*°	*q*_5_*/*°	*q*_6_*/*°	l*/mm
1	− 70.1	17.8	− 3.6	− 15.2	73.1	− 52.6	485.97
2	− 63	17.8	− 3.6	− 15.2	73.1	− 52.6	479.79
3	− 65.4	14.8	− 3.6	− 15.2	73.1	− 52.6	502.39
4	− 71.5	12.5	− 3.6	− 15.2	73.1	− 52.6	525.69
5	− 68.1	13.3	− 3.6	− 15.2	73.1	− 52.6	515.97

**Table 4:** Characteristics of the drawstring.

Item	Specification
Measuring range	2000 mm
Maximum speed	1000 m/s
Extension force	5 N
Resolution	0.004 mm
Temperature range	− 25 ℃ ~ + 85 ℃

**Table 5:** Compared algorithms.

Methods	Description
M1	The Levenberg–Marquardt (LM) algorithm stands as a cornerstone in the field of robot calibration, largely due to its superior computational efficiency. This algorithm elegantly bridges the gap between the Gauss–Newton method and the method of gradient descent, offering a robust approach to solving nonlinear least squares problems that are commonplace in robot kinematics and dynamics^16,19,22^
M2	The Extended Kalman Filter (EKF) is a powerful tool for robot calibration, effectively mitigating measurement noise and improving the precision of robot positioning. By modeling the robot's dynamics and incorporating real-time sensor data, the EKF dynamically adjusts to new measurements, ensuring accurate calibration even in the presence of uncertainty. This makes it invaluable for applications requiring high levels of accuracy and reliability in robotic systems^10^
M3	Particle Filtering (PF) excels in handling non-Gaussian systems, making it a robust choice for robot calibration tasks where the uncertainty does not follow normal distribution patterns. By utilizing a set of particles to represent the distribution of possible states, PF can effectively estimate the state of a robot even in complex, nonlinear environments^15^
M4	The Sine Cosine Algorithm (SCA) leverages the mathematical sine and cosine functions to navigate the search space for solving optimization problems. This approach allows SCA to efficiently explore and exploit the solution space, dynamically adjusting its search strategy based on the position of the best solution found so far^34^
M5	The Quadratic Interpolated Beetle Antennae Search (QIBAS) algorithm represents an advanced approach to robot calibration, building upon the concept of mimicking a beetle's antennae movement to explore the solution space. By incorporating quadratic interpolation into the search mechanism, QIBAS effectively refines its ability to navigate through complex parameter spaces, enabling more precise identification of optimal calibration settings^35^
M6	The Step-Size Levenberg–Marquardt (SSLM) algorithm-based calibration model introduces a dynamic adjustment mechanism for the algorithm's step size, enhancing its efficiency and accuracy in solving calibration problems. This modification allows the SSLM algorithm to adaptively fine-tune its approach based on the specific characteristics of the calibration task at hand^19^
M7	The proposed LTLM algorithm, which can effectively enhance the calibration accuracy

#### Performance comparison

**Figure 6:**
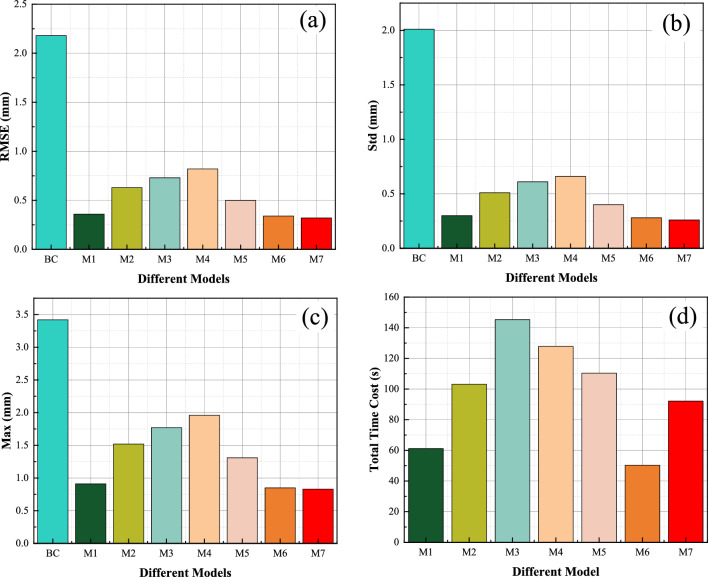
Performance comparison. (**a**) RMSE; (**b**) SD; (**c**) Max; (**d**) Time. Note that BC is the abbreviation for before compensation.

**Figure 7:**
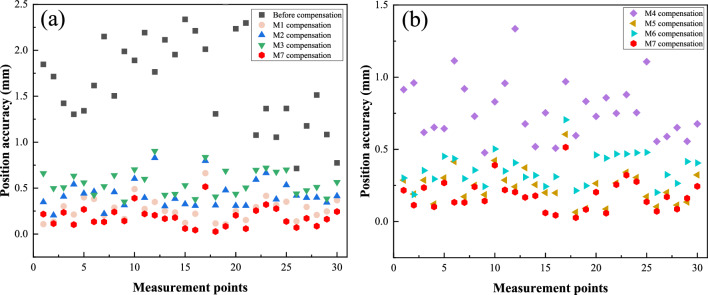
The position accuracy after compensation by methods. (**a**) BC, M1, M2, M3 and M7; (**b**) (**a**) M4, M5, M6 and M7.

**Figure 8:**
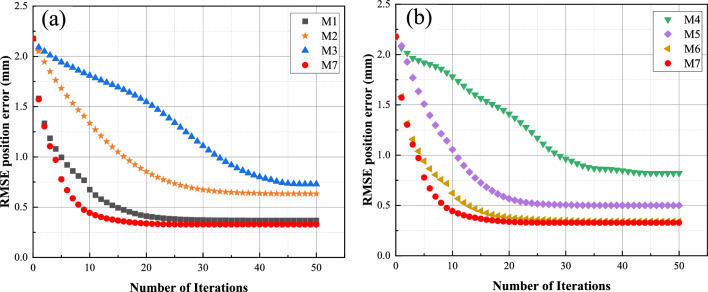
Comparison of convergence curves. (**a**) M1, M2, M3 and M7; (**b**) M4, M5, M6 and M7.

To validate the effectiveness of the proposed method, we compared it with several state-of-the-art calibration methods. Table 6 presents the RMSE, Mean, and MAX values of the compared methods. The geometric parameters obtained after applying M5 compensation are listed in Table 7. Figure 6 displays the performance of compensation using these methods, while Fig. 7 illustrates the compensation accuracy achieved by them. Additionally, Fig. 8 presents the training curves of the methods. From these experimental results, we derived the following crucial findings:

- The LTLM method demonstrated a substantial improvement in compensation accuracy. Figure 6a–c and Table 6 present the results, showing that the RMSE, Mean, and Max values for M7 are 0.32, 0.26, and 0.83, respectively. These values are considerably lower compared to the values of 2.18, 2.01, and 3.42, respectively, before compensation. Specifically, the compensation accuracy achieved by LTLM resulted in reductions of 85.32%, 87.06%, and 75.73% for RMSE, Mean, and Max, respectively. This highlights the significant improvement achieved by the LTLM method.
- M7 exhibits the highest compensation accuracy among M1 ~ M6. Figure 6a–c and Table 6 illustrate that M7 has an RMSE of 0.32, a Mean of 0.26, and a MAX of 0.83. In contrast, M6, which is its closest competitor, has an RMSE of 0.34, a Mean of 0.28, and a MAX of 0.85, resulting in accuracy improvements of 6%, 7.14%, and 2.35%, respectively. Therefore, the proposed method effectively enhances the accuracy of grinding robot compensation.
- Figure 8 illustrates that M7 has the fastest convergence rate. It converges in RMSE after only 20 iterations, whereas M6 takes 30 iterations to converge in RMSE. Therefore, integrating logistic-tent chaotic mapping into the updating rule of the Levenberg Marquardt algorithm significantly enhances its convergence rate.
- The LTLM incurs a higher time cost compared to the standard LM algorithm, yet it remains lower than the majority of compensation methods. As shown in Fig. 6d, the time cost of the proposed LTLM model, M7, is higher than that of M1 and M6 but generally lower than that of M2-M5. This can be attributed to the additional time required by the logistic-tentative chaotic mapping for generating candidate solution sets and evaluating their fitness values. Despite the extended time investment, the justifiable trade-off between higher compensation accuracy and an acceptable time cost is worthwhile.
- In this study, we employ M7 to compensate the geometric parameters of the robot, and the compensation result is depicted in Fig. 6. Furthermore, by comparing the positioning error of the robot after compensation with these methods (as illustrated in Fig. 7), the experimental results demonstrate a significant improvement in the positioning accuracy of the robot.

**Table 6:** Position error.

Model	RMSE (mm)	SD (mm)	Max (mm)
BC	2.18	2.01	3.42
M1	0.36	0.30	0.91
M2	0.63	0.51	1.52
M3	0.73	0.61	1.77
M4	0.82	0.66	1.96
M5	0.50	0.40	1.31
M6	0.34	0.28	0.85
M7	0.32	0.26	0.83

**Table 7:** Parameters after compensation with the LTLM.

Joint *i*	*a*_*i*_*/*mm	*d*_*i*_*/*mm	*α*_*i*_*/*deg	*θ*_*i*_*/*deg
1	− 0.490	289.509	− 89.216	2.315
2	270.031	0.058	3.211	− 92.742
3	69.332	0.253	− 91.915	3.247
4	− 0.653	301.231	88.273	1.346
5	− 0.611	0.181	− 91.374	− 4.218
6	− 0.108	72.416	0	1.546

In conclusion, the analysis results unequivocally establish that LTLM has attained a remarkable level of compensation accuracy, surpassing its peers in terms of effectiveness.

## Grinding compensation experiment

Robots are extensively employed in the component manufacturing domain for the tasks of grinding in ultra-precision components. The grinding process of components through a robot entail following a predefined path dictated by the NC program. However, the presence of geometric error in the robot introduces absolute positioning deviations, resulting in deviations between the actual processing path and the desired theoretical processing path. These deviations have a profound impact on the overall quality of the components being processed^36^.

To validate the efficacy of the proposed compensation method in enhancing the grinding accuracy of components surface, a simulation-based grinding experiment is conducted. Initially, a component is carefully selected, Subsequently, the NC code is generated by utilizing the surface equation specific to the component, yielding the corresponding machining path points. Notably, these path points represented the theoretically ideal paths, devoid of any deviations.

During the simulation grinding experiment, positioning error are deliberately introduced into the theoretical path prior to the application of robot compensation, specifically for M1 and M7 compensation methods. This process resulted in two distinct sets of paths: the before compensated grinding path, the grinding path compensated by M1 ~ M7. These paths are subsequently employed to conduct separate simulation grinding experiments.

The results of the simulation grinding experiments are presented in Fig. 8. For evaluating accuracy, five points are selected in each scenario, with the results presented in Table 8.

**Table 8:** Deviation of path points on surfaces under different models.

Models	Deviation of each point (mm)					Mean	SD
	P1	P2	P3	P4	P5		
BC	1.83	3.42	1.65	− 0.95	0.62	1.31	1.62
M1	0.91	0.24	0.14	0.49	0.40	0.44	0.30
M2	0.89	0.80	0.06	0.57	1.25	0.71	0.44
M3	1.77	1.28	0.89	1.09	0.75	1.15	0.40
M4	0.97	1.80	1.44	0.67	0.52	1.08	0.53
M5	0.68	0.20	− 0.44	1.31	1.28	0.61	0.74
M6	0.27	− 0.01	0.45	0.51	0.65	0.37	0.26
M7	0.16	0.38	0.32	− 0.10	0.15	0.18	0.19

**Figure 9:**
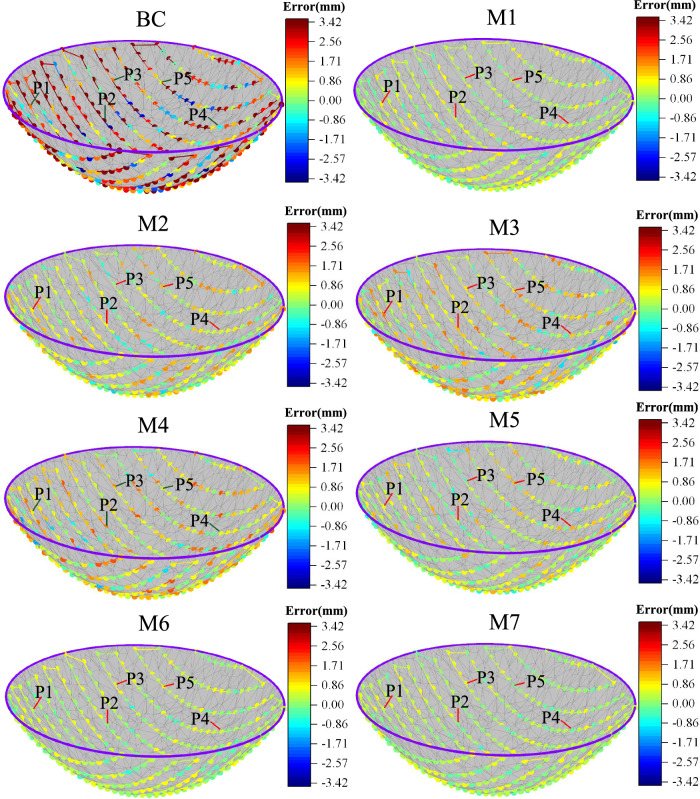
Comparison of grinding compensation path results.

From Fig. 9 and Table 8, we can discern several notable findings:

1) The LTLM compensation method (M7) has significantly improved the grinding path deviation compensation. As depicted in Fig. 9 and Table 8, the mean and standard deviation for M7 is 0.18 and 0.19, respectively. Compared to the before compensation, there is a decrease of 86.26% in the mean and a decrease of 88.27% in the standard deviation.

2) M7 achieves the lowest grinding path deviation among the models evaluated. As depicted in Fig. 9 and Table 8, specifically, the mean deviation of M7 is lower than that of the other models (M1 through M6) by 59.09%, 74.65%, 84.35%, 83.33%, 70.49%, and 51.35%, respectively. For the standard deviation, M7 shows a reduction compared to M1 through M6 by 36.67%, 56.82%, 52.5%, 64.15%, 74.32%, and 26.92%, respectively.

In summary, the LTLM compensation method embodied by model M7 demonstrates a significant improvement in the grinding path accuracy of components. Figure 9 illustrates that M7 achieves the minimum deviation in path points. The processing path and path points of M7 are closer in color to the 0 area, indicating minimal deviation. These results unequivocally indicate that LTLM effectively enhances the surface path points accuracy of the components, providing substantial benefits over the before compensation state and comparative models M1 through M6.

## Conclusion

In this study, we introduce the LTLM method, an innovative approach to geometric error compensation in grinding robots, aimed at significantly improving the accuracy of machined components. Our findings demonstrate that LTLM notably enhances the absolute positioning accuracy of grinding robots, with an 85.32% improvement in RMSE post-compensation. Compared to existing methods like LM, EKF, PF, SCA, QIBAS, and SSLM, LTLM shows substantial superiority, underscoring its effectiveness and potential as a benchmark in robotic calibration accuracy.

The LTLM method significantly reduces geometric errors in grinding robot operations, as evidenced by simulation-based experiments. This reduction in errors directly translates to improved machining accuracy and component quality. At the heart of LTLM is the use of logistic-tent chaotic mapping for the iterative construction of candidate solutions, enhancing identification accuracy and setting a new approach in calibration processes.

In future research, our primary focus will be on refining the computational architecture of the LTLM method to enhance its computational efficiency. This will facilitate rapid identification and compensation of geometric errors in robotic systems, streamlining the calibration process and significantly improving the precision and reliability of robotic operations.

## Data availability 

Data underlying the results presented in this paper are available in Dataset: https://github.com/Lizhibing1490183152/RobotCali.

## References

[1] Li Z, Li S, Luo X (2021). An overview of calibration technology of industrial robots. IEEE/CAA Journal of Automatica Sinica.

[2] Xu K, Xu S, Qi Q (2023). Research on high-precision positioning method of robot based on laser tracker. Intel. Serv. Robot..

[3] Hu M, Wang H, Pan X, Liao L, Sun H (2022). Elastic deformation modeling of series robots with consideration of gravity. Intel. Serv. Robot..

[4] Petrič T, Žlajpah L (2023). Kinematic model calibration of a collaborative redundant robot using a closed kinematic chain. Sci. Rep..

[5] Qian W, Song S, Liu K, Zeng X, Yin X, Xie L (2022). Motion error analysis of a shield machine tool-changing robot based on a screw-vector method. Sci. Rep..

[6] Wu L, Yang X, Chen K, Ren H (2015). A minimal POE-based model for robotic kinematic calibration with only position measurements. IEEE Trans. Autom. Sci. Eng..

[7] Stone, H. & Sanderson, A. Statistical performance evaluation of the s-model arm signature identification technique. In *Proceedings. 1988 IEEE International Conference on Robotics and Automation*, 2, 939–946(1988).

[8] Nubiola A, Bonev I (2013). Absolute calibration of an ABB IRB 1600 robot using a laser tracker. Robotics and Computer-Integrated Manufacturing.

[9] Ma L, Bazzoli P, Sammons P, Landers R, Bristow D (2018). Modeling and calibration of high-order joint-dependent kinematic errors for industrial robots. Robotics and Computer-Integrated Manufacturing.

[10] Deng Y, Hou X, Li B, Wang J, Zhang Y (2024). A highly powerful calibration method for robotic grinding system calibration via using adaptive residual extended Kalman filter. Robotics and Computer-Integrated Manufacturing.

[11] Li X, Zhang E, Fang X, Zhai B (2022). Calibration Method for Industrial Robots Based on the Principle of Perigon Error Close. IEEE Access.

[12] Zhu Q, Xie X, Li C, Xia G, Liu Q (2019). Kinematic Self-Calibration Method for Dual-Manipulators Based on Optical Axis Constraint. IEEE Access.

[13] Le, P. & Kang, H. A Robotic Calibration Method Using a Model-Based Identification Technique and an Invasive Weed Optimization Neural Network Compensator. *Applied Sciences-Basel,* 10(20), 7320(2020).

[14] Yan Y (2020). Error recognition of robot kinematics parameters based on genetic algorithms. J. Ambient. Intell. Humaniz. Comput..

[15] Jiang Z, Zhou W, Li H, Mo Y, Ni W, Huang Q (2018). A new kind of accurate calibration method for robotic kinematic parameters based on the extended Kalman and particle filter algorithm. IEEE Trans. Industr. Electron..

[16] Deng Y, Hou X, Li B, Wang J, Zhang Y (2023). A Novel Positioning Accuracy Improvement Method for Polishing Robot Based on Levenberg–Marquardt and Opposition-based Learning Squirrel Search Algorithm. J. Intell. Rob. Syst..

[17] Bastl P, Chakraborti N, Valášek M (2023). Evolutionary algorithms in robot calibration. Mater. Manuf. Processes.

[18] Chen X, Zhan Q (2022). The Kinematic Calibration of an Industrial Robot with an Improved Beetle Swarm Optimization Algorithm. IEEE Robotics and Automation Letters.

[19] Li Z, Li S, Luo X (2023). Efficient Industrial Robot Calibration via a Novel Unscented Kalman Filter-Incorporated Variable Step-Size Levenberg–Marquardt Algorithm. IEEE Trans. Instrum. Meas..

[20] Xu X, Bai Y, Zhao M, Yang J, Pang F, Ran Y, Tan Z, Luo M (2023). A Novel Calibration Method for Robot Kinematic Parameters Based on Improved Manta Ray Foraging Optimization Algorithm. IEEE Trans. Instrum. Meas..

[21] Deng Y, Hou X, Li B, Wang J, Zhang Y (2023). A novel method for improving optical component smoothing quality in robotic smoothing systems by compensating path errors. Optical. Express.

[22] Li, Z., Li, S., Bamasag, O., Alhothali, A. & Luo, X. Diversified Regularization Enhanced Training for Effective Manipulator Calibration. IEEE Trans Neural Netw Learn Syst, doi: 10.1109/TNNLS.2022.3153039. (2022) 10.1109/TNNLS.2022.3153039 35263261

[23] Zhao H, Yu L, Jia H, Li W, Sun J (2016). A New Kinematic Model of Portable Articulated Coordinate Measuring Machine. Applied Sciences.

[24] Wu L, Crawford R, Roberts J (2017). An Analytic Approach to Converting POE Parameters Into D-H Parameters for Serial-Link Robots. IEEE Robotics and Automation Letters.

[25] Huang B, Ma C (2019). A Shamanskii-like self-adaptive Levenberg–Marquardtt method for nonlinear equations. Computers & Mathematics with Applications.

[26] Nielsen H (1999). Damping Parameter in Marquardtt's Method.

[27] Li X, Wang J, Hao W, Zhang M, Wang M (2022). Chaotic arithmetic optimization algorithm. Applied Intelligence.

[28] Zhang X, Feng T (2018). Chaotic bean optimization algorithm. Soft Computing.

[29] Wang Y, Zhang Q, Wang G, Hu Z (2022). An enhancing many-objective evolutionary algorithm using chaotic mapping and solution ranking mechanism for large-scale optimization. Journal of Computational Design and Engineering.

[30] Ma M, Wu J, Shi Y, Yan L, Lu W (2022). Research on Multiaircrafts Cooperative Arraying to Jam Based on Multiobjective Moth-Flame Optimization Algorithm. IEEE access.

[31] Yusof N, Muda A, Pratama S, Carbo-Dorca R, Abraham A (2022). Improving Amphetamine-type Stimulants drug classification using chaotic-based time-varying binary whale optimization algorithm. Chemometrics and Intelligent Laboratory Systems.

[32] Ma M, Wu J, Shi Y, Yue L, Yang C, Chen X (2022). Chaotic Random Opposition-Based Learning and Cauchy Mutation Improved Moth-Flame Optimization Algorithm for Intelligent Route Planning of Multiple UAVs. IEEE access.

[33] Zhang F, Shang W, Li G, Cong S (2021). Calibration of geometric parameters and error compensation of non for cable-driven robots. Mechatronics.

[34] Mirjalili S (2015). SCA: A Sine Cosine Algorithm for solving optimization problems. Knowledge-Based Systems.

[35] Li Z, Li S, Luo X (2022). Using Quadratic Interpolated Beetle Antennae Search to Enhance Robot Arm Calibration Accuracy. IEEE Robotics and Automation Letters.

[36] Deng Y, Hou X, Li B, Wang J, Zhang Y (2023). Review on mid-spatial frequency error suppression in components manufacturing. International Journal of Advanced Manufacturing Technology.

